# When to start antiretroviral therapy: the need for an evidence base during early HIV infection

**DOI:** 10.1186/1741-7015-11-148

**Published:** 2013-06-14

**Authors:** Jens D Lundgren, Abdel G Babiker, Fred M Gordin, Álvaro H Borges, James D Neaton

**Affiliations:** 1 Department of Infectious Diseases, Rigshospitalet, Copenhagen, Denmark; 2 Copenhagen HIV Programme, University of Copenhagen, The Panum Institute/Building 21.1, Blegdamsvej 3B, 2200, Copenhagen, N, Copenhagen, Denmark; 3 MRC Clinical Trials Unit, Aviation House, 125 Kingsway, London WC2B 6NH, UK; 4 Veterans Affairs Medical Center and George Washington University, 50 Irving St, Washington, DC 20422 202-745-8000, USA; 5 Division of Biostatistics, University of Minnesota, A460 Mayo Building, 420 Delaware Street, Minneapolis, MN 55455, USA

**Keywords:** Antiretroviral therapy, Benefit, CD4 count, Drug toxicity, HIV, Risk ratio, Treatment as prevention

## Abstract

**Background:**

Strategies for use of antiretroviral therapy (ART) have traditionally focused on providing treatment to persons who stand to benefit immediately from initiating the therapy. There is global consensus that any HIV+ person with CD4 counts less than 350 cells/μl should initiate ART. However, it remains controversial whether ART is indicated in asymptomatic HIV-infected persons with CD4 counts above 350 cells/μl, or whether it is more advisable to defer initiation until the CD4 count has dropped to 350 cells/μl. The question of when the best time is to initiate ART during early HIV infection has always been vigorously debated. The lack of an evidence base from randomized trials, in conjunction with varying degrees of therapeutic aggressiveness and optimism tempered by the risks of drug resistance and side effects, has resulted in divided expert opinion and inconsistencies among treatment guidelines.

**Discussion:**

On the basis of recent data showing that early ART initiation reduces heterosexual HIV transmission, some countries are considering adopting a strategy of universal treatment of all HIV+ persons irrespective of their CD4 count and whether ART is of benefit to the individual or not, in order to reduce onward HIV transmission. Since ART has been found to be associated with both short-term and long-term toxicity, defining the benefit:risk ratio is the critical missing link in the discussion on earlier use of ART. For early ART initiation to be justified, this ratio must favor benefit over risk. An unfavorable ratio would argue against using early ART.

**Summary:**

There is currently no evidence from randomized controlled trials to suggest that a strategy of initiating ART when the CD4 count is above 350 cells/μl (versus deferring initiation to around 350 cells/μl) results in benefit to the HIV+ person and data from observational studies are inconsistent. Large, clinical endpoint-driven randomized studies to determine the individual health benefits versus risks of earlier ART initiation are sorely needed.

The counter-argument to this debate topic can be freely accessed here: http://www.biomedcentral.com/1741-7015/11/147.

## Background

Since the first transmissions from chimpanzees to men almost a century ago [1], HIV continues to spread endemically (and in some regions still epidemically) throughout the world. Most transmissions occur during sex, whereas mother-to-child transmission constitutes one-eighth of the annual 2.5 million new infections [2]. Use of unclean needles among injecting drug communities also contributes to rapid spread which in Eastern Europe allowed for a secondary wave of sexual transmission [3].

The prospect of an effective HIV vaccine to contain the pandemic remains uncertain [4]. Effective interventions to reduce risk of transmission are diverse and include consistent use of condoms, circumcision of uninfected men (for acquisition of HIV through heterosexual sex) [5,6], and use of clean needles for injection of drugs [7]. Although these interventions, when used effectively and widespread have contained the spread, they are unlikely to be able to eliminate the HIV pandemic. Hence, other effective interventions are urgently needed.

In 1994, the first evidence emerged to suggest that lowering the replication of HIV in the infected host by use of antiretroviral therapy (ART) reduced the risk of transmission [8]. Pregnant women placed on zidovudine transmitted HIV less frequently to their offspring. This concept was subsequently simplified (for example, single-dose nevirapine and use of a standard ART regimen), and if used optimally can virtually eliminate mother-to-child transmission. Although this intervention is available in most places in the world, 330,000 infants still become infected each year [2]. This underlines the fact that major barriers remain within health systems that limit real life implementation of even this efficacious and relatively simple intervention.

In 2011, it was conclusively shown that lowering viral replication in the HIV-infected person also reduces heterosexual transmission [9]. Major policy changes have since then been made based on this observation to dampen global spread of HIV, with the prospect of having more success than has been accomplished for mother-to-child transmission.

Left untreated, most infected persons will eventually die typically from opportunistic complications due to the resulting immunodeficiency. ART has saved millions of lives [10,11] after the groundbreaking discovery in 1997 that the duration of effect could be extended by combining three antiretroviral drugs [12]. However, ART does not eradicate HIV and, once ART is initiated, it must be used lifelong without interruption [13]. Investment in ART treatment programs hence require long-term commitment from the infected person and the health system. World Health Organization (WHO)-led efforts to ensure universal access to ART have resulted in an accelerated introduction of ART in resource-limited settings [14]. Nearly half of the funding for this is provided by donor countries, although the global fund that manages this process has recently suffered from a shortage of pledged funds, likely due to the global financial crisis over the last several years.

Like any medication, ART is associated with adverse effects. Thus, strategies for use of ART have focused on providing treatment to persons who stand to benefit immediately from initiating the therapy. HIV infection progresses slowly in most persons, and life-threatening complications in untreated persons usually only start to emerge several years after the inception of the infection. The number of circulating CD4+ lymphocytes, the primary target for HIV, is the best laboratory marker of HIV-induced immune damage. There is global consensus that any HIV+ person with less than 350 CD4+ lymphocytes per μl blood should initiate ART, as should HIV+ persons with clinical HIV-related complications of a certain severity regardless of their CD4 count [15-19].

Conversely, it remains controversial whether ART is indicated in asymptomatic HIV-infected persons with CD4 counts above 350 cells/μl or whether it is more advisable to defer initiation until the CD4 count has dropped to 350 cells/μl or less. The arguments for and against such ‘early’ (in the course of HIV infection) use of ART are reviewed below. A divergent expert view has also been published in *BMC Medicine *[20].

## Discussion

### Characteristics of and challenges in diagnosing persons newly infected with HIV

In regions with concentrated epidemics, average age at infection is typically around 30 or slightly higher, and men tend to be more affected than women. Conversely, in areas with a generalised epidemic, the age at infection tends to be younger and young women are disproportionately affected [2].

Most newly infected persons remain undiagnosed. The testing strategies used in the last two decades remain ineffective in diagnosing a large percentage of asymptomatic persons early in the course of their infection. Consequently, 50% or more of HIV+ persons present to care late in the course of their infection and with CD4 counts below 350 cells/μl [21,22]. Late presentation for care leads to excess mortality despite initiation of ART [23,24], higher cost to health systems [25], and is a missed prevention opportunity [26].

Better and more innovative HIV testing strategies are urgently needed to address this major public health challenge [27]. HIV tests were first used in 1985 [28], but their strategic use to identify persons earlier in the course of their infection has been remarkably slow. This is in contrast to the rapid evolution in knowledge on use of ART and the significant current push to use it early. In large part, the latter reflects support from the pharmaceutical industry.

### Evidence of benefits from early use of ART

Data from three randomized trials [9,29,30] and from four observational studies [31-34] support the use of ART when the CD4 count declines to 350 cells/μl. The entry CD4 criteria and the deferral strategy of the participants in the three randomized trials are summarized in Table 1.

**Table 1:** Summary of baseline characteristics and deferral strategies from randomized controlled trials comparing deferred vs immediate initiation of antiretroviral therapy (ART) in ART-naïve HIV+ persons

**Study (reference)**	**Sample size**	**Median baseline CD4 count (cells/μl)**	**Deferral strategy**	**Median CD4 count (cells/μl) at ART initiation in the deferred arm**
SMART [29]	249	437	ART deferred until:	245
			1. CD4 declined to <250 cells/μl	
			2. CD4 percentage declined to <15%	
			3. Symptoms of HIV disease developed	
CIPRA HT-001 [30]	816	281	ART deferred until:	166
			1. CD4 declined to ≤200 cells/μl	
			2. AIDS-defining illness developed	
HPTN 052 [9]	1,761	428	ART deferred until:	299
			1. CD4 declined to ≤250 cells/μl	
			2. AIDS-defining illness developed	

A systematic review by the Cochrane Collaboration was carried out in 2011 [35] before the findings of HIV Prevention Trials Network study 52 (HPTN 052) were announced [9,36]. The authors of this review pooled data from the trial in Haiti [30] and the Strategies for Management of Anti-Retroviral Therapy (SMART) subgroup [29]. They reported that the risk of death was reduced by 74% (relative risk (RR) = 0.26; 95% CI 0.11 to 0.62; *P* = 0.002). They concluded that ‘there is evidence of moderate quality that initiating ART at CD4 levels higher than 200 or 250 cells/μl reduces mortality rates in asymptomatic ART-naïve, HIV-infected people’ [36]. These data and the data on mortality for HPTN 052 [9] are summarized in Table 2. The pooled RR for death with inclusion of HPTN 052 is 0.43 (95% CI 0.24 to 0.77; *P* = 0.003). The pooled data from the Haiti trial and HPTN 052 also indicated that risk of tuberculosis (TB) is decreased by 49% (95% CI 0.34 to 0.76) with earlier treatment [9,30]. In the SMART subgroup there were no cases of TB reported [29]. Therefore, all three of these trials are providing evidence that ART should be initiated at CD4 counts higher than 250 cells/μl since the deferral strategy was consistently to only initiate ART when the CD4 count dropped to levels below this threshold. In our opinion, the pooled evidence of trials with the inclusion of HPTN 052 does not change the conclusions of the earlier meta-analysis: the evidence remains moderate that ART should be initiated at CD4 counts higher than 250 cells/μl based on the data from 3 trials in which a total of 53 deaths occurred.

**Table 2:** Impact of immediate vs deferred initiation of antiretroviral therapy (ART) on mortality: data from randomized controlled trials involving ART-naïve HIV+ persons

**Study (reference)**	**Deaths, early ART initiation**^**a**^**arm**	**Deaths, deferred ART initiation**^**b**^**arm**
SMART [29]	0/131	1/118
CIPRA HT-001 [30]	6/408	23/408
HPTN 052 [9]	10/886	13/877
Pooled data from the three trials	16/1,425	37/1,403

^a^Early ART initiation defined as start of ART at CD4 >350 cells/μl.

^b^Deferred ART initiation defined as start of ART at CD4 <200 or <250 cells/μl.

The recommendation to begin ART when the CD4 count declines to 350 cells/μl comes from observational studies. In this regard, the four observational studies are consistent. For example in Cain *et al*. [34], hazard ratios (HRs) for AIDS or death were 1.37 (95% CI 1.20 to 1.57) and 1.20 (95% CI 1.05 to 1.38) for CD4 thresholds of 200 and 250 cells/μl versus 350 cells/μl. Corresponding HRs for death, which were not significant, were 1.18 (95% CI 0.95 to 1.46) and 1.07 (95% CI 0.86 to 1.34). Using the rating system employed by the group writing guidelines for the US Department of Health and Human Services (DHHS) [19] (see below), we believe this recommendation should be rated BII.

There is no evidence from randomized controlled trials to suggest that a strategy of initiating ART when the CD4 count is above 350 cells/μl (versus deferring initiation to around 350 cells/μl) results in health benefit to the HIV+ person. The only direct evidence addressing this question comes from the analyses of the four observational studies, and the results are inconsistent [31-34] and all limited by not having data on serious end organ diseases and cancer. For the question of initiation of ART between 350 to 500 cells/μl (versus deferring to CD4 count <350 cells/μl), for example, two of the articles report an excess risk of death from deferral [31,33], whereas the two others fail to find evidence of such an association [32,34]. For the question of initiation above 500 cells/μl (versus deferring to 350 to 500 cells/μl), one article reported an excess risk of death from deferral [31], whereas two others failed to find evidence of such an association [32,33]. Importantly, the observational studies that these analyses are based on were all conducted in the last 10 to 12 years, and where the approach to initiating ART was relatively conservative. As such, the persons that did indeed start ART early were not representative of the populations in care at the time, making the internal and external validities of the findings to the contemporary discussion questionable. The lack of representativeness is evident from the reported death rates in the North American AIDS Cohort Collaboration on Research and Design (NA-ACCORD) study [31]. The authors report death rates of 1.6 and 1.3 per 100 person years for those initiating ART between 351 and 500 cells/μl and >500 cells/μl, respectively. These rates are much higher than reported in the other observational studies and in large clinical trials [13,37].

There are numerous studies available to suggest that untreated HIV (or ongoing viral replication) may be harmful to the host [13,38-40]. Some experts argue that our contemporary understanding of the pathogenesis linking untreated HIV replication to end- organ diseases and cancer is so evident that earlier ART initiation would be justified without the need to conduct randomized trials [20]. It is beyond the scope of this review article to critique this body of evidence. Collectively, what these studies do show is that advanced and untreated HIV disease is detrimental to health as is intermittent (or ineffective) use of ART versus continued suppressive ART, and that those persons who experienced better CD4 cell recovery will do better than those who do not. In fact, the standardized mortality ratio for the latter group (that is, those able to recover to a CD4 count above 500 cells/μl from typical low nadir CD4 levels (<200 cells/μl)) approaches one [41,42], suggesting that prior severe immunodeficiency may not lead to long-term harm as long as the person recovers a substantive number of CD4+ lymphocytes and hence experiences optimal benefit of ART. When ART is initiated in patients with low nadir CD4 counts, recovery of CD4 cells may be less likely to occur in a given timespan [43], but is likely achievable in most patients if viral suppression is maintained [44]. Recovery of CD4 counts to levels above 500 cells/μl occurs quickly in virtually all patients in case ART is initiated around 350 cells/μl [44]. Importantly, none of these studies address the question focused on in this review, namely as compared to deferral of ART to 350 cells/μl whether initiating ART at a CD4 count higher than 350 cells/μl results in a reduced risk of morbidity and mortality to the participant with chronic HIV infection [45]. A clinical trial comparing early versus later initiation of ART with enough power to detect differences in incidence of end-organ diseases and cancer is required to address that question.

Others argue that it takes such a short time to progress from time of infection to CD4 counts below 500 and 350 cells/μl that it is not worth the bother to delay initiation of ART as the person will be in need shortly anyway. However, in the first 2 years following seroconversion, estimated mean CD4+ count loss has been shown to range broadly from 88 to 167 cells/μl [46]. Therefore, in a considerable fraction of HIV+ persons, a long period encompassing several years or even decades will elapse before the CD4+ count thresholds of 500 and 350 cells/μl are reached.

### Why do different expert groups view these data so differently?

Interpretation of currently available data varies among experts and treatment guidelines groups. For example, the Department of Health and Human Services Guidelines [19] uses the Haiti trial to support their recommendation of starting at 350 cells/μl and the SMART subgroup and the HPTN 052 trial to support initiating ART for individuals with a count between 350 and 500 cells/μl. In their grading scheme ‘A’ is strong evidence, ‘B’ is moderate, ‘C’ is optional, ‘I’ is data from trials, ‘II’ is data from well-designed observational studies and ‘III’ is expert opinion. They rate the evidence as AI for <350 cells/μl; AII for 350 to 500 cells/μl. Their ratings of AI and AII appear to be based on consideration of the entry criteria for the trials and not the deferral strategy. WHO [15], again on the basis of the Haiti and SMART trials, graded the evidence supporting early ART initiation in another way. Overall, the recommendation was to defer ART initiation until CD4 counts decline to <350 cells/μl or an AIDS-defining illness develops (clinical stage 3 or 4, as per WHO clinical staging classification of HIV disease). The recommendation to start ART at CD4 levels between 200 to 250 and 350 cells/μl was considered ‘strong’ by WHO, but the evidence supporting this strategy was considered ‘moderate’.

Earlier use of ART would always show benefit if the deferral strategy was merely to observe the untreated HIV infection with no implementation of therapy. The deferral strategy in all of the quoted randomized controlled trials aimed at starting ART once the CD4 count dropped below 250 cells/μl, although a substantive proportion of persons allocated to this arm were allowed to progress to CD4 count below 200 cells/μl before ART was initiated. In the most recent of these trials, HPTN 052, the median CD4 count at time of initiation of ART in the HIV+ persons randomized to deferring ART was 229 cells/μl and with a interquartile range from 197 to 249 cells/μl, implying that 25% of the persons initiated ART when the CD4 count had dropped below 200 cells/μl [9]. Hence, our view of these randomized trials, as stated above, is that they support starting ART above 250 cells/μl. None of the trials provided evidence for starting ART between 350 and 500 cells/μl.

The DHHS guidelines use observational data to support initiating ART >500 cells/μl and their recommendation is BIII [19]. The rating of ‘III’ seems appropriate, but as noted above, the findings from the four observational studies are inconsistent, so in our opinion the full rating should be CIII, not BIII. The credibility of expert opinion would be enhanced if the authors of the four observational studies reconciled their differences and reported their consensus on the findings. Of note, in the WHO guidelines, no critical appraisal of the currently available data supporting (or not) ART initiation in treatment-naive symptomless HIV+ with CD4 >350 cells/μl was made [15]. No new critical data have been published since the release of the WHO guidelines in 2010, and hence we must assume that the WHO remains committed to this appraisal of the evidence.

### Evidence of harm from early use of ART

Recently introduced antiretroviral drugs have fewer side effects compared to the first and second-generation drugs developed 10 to 15 years ago. However, central nervous system toxicity (possibly linked with depression and suicide) [47-49], renal toxicity (urolithiasis [50] and progressive worsening of kidney function [51,52]), bone toxicity (demineralization) [53], and cardiovascular toxicity (platelet hyper-reactivity linked with excess coronary artery disease and progressive likely accelerated atherosclerosis) [54,55] are known adverse reactions to contemporarily used drugs in resource-rich countries.

It is challenging to conduct research aimed at identifying such adverse drug reactions since many of these events only develop after extended periods of exposure and may be infrequent in the relatively healthy populations allowed to enter short-term trials carried out for drug registration. Therefore, most of the knowledge on the clinical adverse drug effect profile from use of ART is derived from observational studies for which confounding factors limit the ability to reliably establish causal relationships between drug exposure and specific adverse outcomes. It is noteworthy that whereas knowledge and appreciation of benefits from use of an intrinsically effective antiretroviral drug is fairly easily demonstrated in the pivotal trials required by authorities for marketing, the limited sample size and follow-up and the selection of relatively health participants compromises a comprehensive understanding of possible adverse drug reactions. It has hence taken several years from when a drug was first licensed until some of the currently well accepted adverse drug reactions were identified. For example, it took 7 years to establish the causal link between use of stavudine or zidovudine and the development of lipodystrophy [56,57]. As such, it remains uncertain whether the current knowledge we have on profile of adverse drug reactions is comprehensive and complete. Recent studies linking cumulative use of ART with possible excess risk of cancer serves as a reminder of this [58,59].

The hypothesis that early (versus deferred) use of ART results in less risk of these various adverse drug reactions has been proposed [60,61] but never conclusively confirmed in randomized controlled trials. The main issue that makes this impossible to say is that adverse drug reactions are often reflected in organ dysfunction, which untreated HIV (and hence low CD4 count) also accentuates [62-65]. Hence, whether organ dysfunction in patients who initiated ART late in the course of HIV infection is the result of the immunodeficiency or the antiretroviral drugs used is impossible to differentiate. Importantly, evaluation of non-fatal adverse drug reaction risk among persons recently infected and initiating ART is limited since this strategy of using ART has only recently been introduced and since most cohort studies are only collecting mortality data, which is a poor proxy for such reactions.

We believe safety is paramount when interventions are proposed for target populations at low risk of morbidity and mortality. As Geoffrey Rose said with respect to another chronic condition: ‘If a preventive measure exposes many people to a small risk, then the harm it does may readily (…) outweigh the benefits, since these are received by relatively few’ [66].

### Evidence for defining the benefit:risk ratio for early use of ART

Whereas initiation of ART at counts <350 cells/μl provides clear advantages to the HIV+ person, defining the benefit:risk ratio is the critical missing link in the discussion on earlier use of ART. For early ART to be justified, this ratio must favor benefit over risk. An unfavorable ratio would undermine the argument of using early ART [67,68]. Such outcomes would violate the basic principle for use of any type of medicine namely to ‘do no harm’, that is, ‘the doctor should not prescribe medications unless s/he knows that the treatment is unlikely to be harmful’.

Based on current knowledge, it is not unreasonable to hypothesize that ART is harmful to use in early HIV infection. The argument for this is the following. Let us imagine the optimistic (albeit uncertain) assumption that early ART provides benefit to the individual. However, for the person to experience this benefit, the probability of becoming ill without ART has to be real, otherwise there is nothing to be gained by being treated. But as the probability of contracting an AIDS event in early HIV is low, it is more likely that the person would suffer from organ dysfunction or cancer; however, the probability of contracting one of these events is also low in most recently infected HIV+ persons for several reasons. Most importantly, most persons get infected relatively early in life when their risk of these diseases is low. As such, even though one assumes benefit of early ART on organ dysfunction and cancer, the probability of contracting such diseases is low even without ART, and hence many persons would need to be treated for one to benefit. Conversely, the risk of adverse drug reactions is real and will occur irrespective of the person’s age. Newer drugs used contemporarily lead to less risk of adverse drug reactions, and as such many need to be treated for one to be harmed. But if the number needed for one to be harmed [69] is higher than the number needed to benefit, early ART is of net harm. If this hypothesis is shown to be correct, this would have major implications not only for future treatment strategies, but also for those that have already started ART early, as it is not advisable to interrupt ART once initiated [13].

Ongoing research aims to clarify the benefit:risk ratio of early ART. The Strategic Timing of Anti-Retroviral Treatment (START) study randomizes asymptomatic HIV+ persons with CD4 count above 500 cells/μl to immediate versus deferred (when CD4 count drops to 350 cells/μl) initiation of ART. The study has already included 4,154 patients and the final sample size of 4,600 is projected to be enrolled by the end of 2013. The study is endpoint driven: the primary endpoint is AIDS, organ disease or cancer [70]. The required 213 primary endpoints are expected to have developed in this cohort by 2016; an estimate reaffirmed recently by the protocol leadership when decision on final sample size was made.

### Special issues in areas with high tuberculosis (TB) endemicity

In untreated advanced HIV, the risk of active TB in *M. tuberculosis* infected persons is 20-fold higher than in the background population [71]. Universal initiation of ART in persons with less than 350 cells/μl will lead to reduced individual morbidity and mortality and to less transmission of TB. In support of this, a meta-analysis published in 2012 found that earlier use of ART reduced risk of contracting tuberculosis disease [72]. The HPTN 052 study, conducted in high TB endemic regions, confirmed that deferral of ART to less than 250 cells/μl (versus starting above 350 cells/μl) leads to excess risk of tuberculosis, although this benefit was surprisingly seen for presumptive extrapulmonary TB events only and not for the more frequently occurring pulmonary TB [9,36]. Conversely, since none of the studies included in the meta-analysis assessed a deferral strategy of initiating ART when the CD4 count approached 350 cells/μl (as such a strategy is not adopted yet in the resource constrained areas where TB is highly endemic), the meta-analysis is unable to shed light on whether earlier use is of benefit to the individual’s health and to reduce forward transmission of the bacteria within the population. In this respect, it is important to be reminded of one of Muench’s postulates namely that ‘nothing improves the performance of therapy like the weakness of controls in its appraisal’ [73].

### Treatment as prevention (TasP)

Some countries are considering adopting a strategy of universal treatment of all HIV+ persons irrespective of their CD4 count, and irrespective of whether they stand to receive personal net benefit, to reduce the infectiousness of the population and hence dampen transmission [74-77]. Although the number of ongoing HIV transmissions remains excessive and novel interventions are attractive to consider, such a strategy is controversial to implement outside of a research setting for several reasons. First, as discussed above, it remains uncertain whether early use of ART is of net benefit to the person commencing ART. It is controversial to assume that the benefit of reducing HIV transmission by earlier ART initiation is equivalent to personal health benefit. Second, the public health concerns with using ART as the primary public health intervention is that this may lead to a perception in the population that other effective preventive measures are no longer required. In communities of men who have sex with men (MSM), such a ‘sexual disinhibition phenomena’ has been observed in the last decade in studies from Western Europe [78-80] and in the USA [81]. Third, a public health strategy of using ART to reduce transmission can only be envisioned to become effective if the infection is diagnosed very early; 30% to 50% of transmissions occur during the first few months after the initial infection [80,82] when viral replication is most extensive [83,84]. Finally, several large population studies are underway to examine the efficacy of TasP [85], and it would appear reasonable to await the results of such trials and the START study before implementing such a strategy.

### Special issues in limited resource settings

The number of new infections is twice as high as the number of persons initiating ART each year. Hence, a continued large number of persons in urgent need of ART (estimated to be 11 million) are not currently getting this life-saving medication [2]. Most of these persons live in resource-limited countries. Introducing a strategy of early use of ART (as part of, for example, a TasP strategy) hence may distract resources and focus from the sectors of the population in most urgent need. Additionally, potential harm from use of early ART in such settings is higher than is the case in more well-resourced countries for two principal reasons. First, older, more toxic drugs (which tend to be cheaper to produce) are preferentially used [86,87]. Second, the proportion of recent infections being caused by a virus resistant to one or more of the preferred initial components of ART is increasing in resource-limited settings and the possibilities for pretreatment resistance testing is very limited [88]. Initiation of ART composed of drugs where the virus is resistant to one or more of the drugs provide suboptimal treatment benefit. Conversely, in settings with very limited access to determining CD4 counts on a regular basis, it could be argued that persons approaching the cut-off of 350 cells/μl (for example, between 350 to 500) are initiated on ART, as deferral without regular CD4 monitoring is potentially hazardous.

### Advice from guidelines

Advice from guidelines may either be based on evidence or based solely on expert opinion. It is noteworthy that different guidelines, released at the same time and with access to the same data, have come out with dissimilar advice on when in the course of HIV, the individual person stands to gain net benefit from initiating ART [67,68]. Some, like the US DHHS [19] and the International Antiviral Society-USA (IAS-USA) guidelines [16], state that ART is beneficial irrespective of the HIV+ person’s CD4 count, whereas for example the British HIV Association guidelines do not recommend use in asymptomatic persons with CD4 counts above 350 cells/μl, but instead call for additional research to address this [17]. Likewise, the European AIDS Clinical Society’s guidelines stresses that there is clinical equipoise for early or deferred initiation above 350 cells/μl as evidence is weak [18]. Advice based primarily on expert opinion has played a major role in HIV medicine over the last two decades, and several of these recommendations have subsequently not been supported by solid evidence and hence abandoned. More generally, it has been pointed out by other authors that reversals of established practices in many fields are common [89]. This emphasizes the importance of large trials to obtain good evidence. For example, many HIV experts recommended intermittent use of ART; indeed, some modestly sized studies declared the approach to be ‘safe’, until the SMART study found in 2006 that such a strategy was harmful [13]. As such, it would appear reasonable to view guidance based on expert opinion skeptically.

### Strategic use of ART versus ART use by physician discretion

The critique of early use of ART outlined above focuses on the strategic use of such a strategy. For the reasons mentioned above, such a strategic use is problematic as there is insufficient evidence to support it and may lead to more harm than benefit to some of those recommended to initiate ART. Conversely, care for individual persons may (and should be allowed to) lead to initiation of early ART, provided that the HIV+ person is well counseled on the lack of evidence, the potential for net harm, that stopping ART subsequently may lead to even more harm and that the decision is made in respect for this person’s rights for personal autonomy and not unduly influenced by his/her loved ones or the health professional s/he consults.

The decision to initiate ART in symptomless patients with early HIV infection is nuanced and each case has to be evaluated on an individual basis. CD4 cell counts thresholds are an important, albeit not absolute or exclusive, parameter upon which such decision should be based. Indeed, from a biological point of view, it makes more sense to take into account ranges of CD4 cell counts, as well as the individual speed of decline of CD4 cell counts, instead of rigid, predetermined thresholds. Finally, even in the hypothetical scenario of a well-documented favorable risk:benefit ratio for initiating ART in early HIV infection, sound clinical judgment, in conjunction with patient willingness and commitment to initiate a lifelong therapy, will remain a crucial step in this shared decision-making process.

### Use of ART in primary HIV infection

Two important studies were recently published focusing on use of ART during primary infection [90,91]. These studies consistently demonstrated that ART can prevent deterioration of the immune system of the HIV+ person that otherwise is seen in persons who remain off ART during and after primary infection. The studies are encouraging, but relevant for individuals with primary infection only; this is a group very challenging to identify in primary care. Conversely, the studies do not address whether those initiating ART during primary infection had clinical benefit from doing so (in terms of reduced morbidity and mortality as the sample in these studies were <1,000 and hence not powered to address this question) and conversely whether allowing persons to progress to lower levels of CD4 count result in any appreciable negative consequences short or long term. Only studies appropriately powered to assess clinical endpoints, requiring substantially larger sample sizes than were available in these two studies, will be able to address these outstanding questions.

## Summary

When to initiate ART in the course of HIV infection has been debated ever since the first drug was introduced in routine care in 1986. ART contemporarily used is effective and reasonably safe. There is global consensus that the benefits: risk ratio favors use of ART in any HIV+ person with moderate HIV-induced immunodeficiency or whom suffers from severe HIV complications, whereas current evidence makes it uncertain whether this ratio is also favorable if ART is initiated earlier on the course of HIV infection. Strategic use of ART in such situations should be avoided until ongoing research efforts have been completed.

## Abbreviations

AIDS: Acquired immunodeficiency syndrome; ART: Antiretroviral therapy; CIPRA HT-001: The comprehensive international program of research on AIDS (CIPRA) HT-001 trial; HIV: Human immunodeficiency virus; HPTN 052: HIV Prevention trials network study 052; HR: Hazard ratio; IAS-USA: International antiviral society-USA; RR: Relative risk; SMART: ‘Strategies for management of anti-retroviral therapy’; START: ‘Strategic timing of antiretroviral treatment’; TB: Tuberculosis; WHO: World Health Organization.

## Competing interests

JDL, JDN, AGB and FMG are co-chairs of START. AHB is the medical officer of the INSIGHT Copenhagen International Coordinating Centre. All authors are members of INSIGHT, a NIH-funded global network for the conduct of trials in infectious diseases including START, which JDN is principal investigator of.

## Authors’ contributions

JDL wrote the first version of the manuscript based on discussions and dialog with the other co-chairs of the START study (FMG and AGB) and the INSIGHT principal investigator (JDN). AHB was primarily responsible for revising the manuscript based on his own perspectives and comments provided by the co-authors. All authors were involved in revising the manuscript and approved the final version prior to submission.

## Authors’ information

JDL, FMG and AHB are all physician-scientists and specialists in infectious diseases. JDL and FMG have practised HIV medicine for more than two decades, and AHB is currently conducting his PhD. AGB and JDN both have a statistical background and have supervised large clinical studies in HIV medicine for the last two decades. This work was presented by JDL, in part, at the HIV 11 congress in Glasgow, UK, November 2012.

## Pre-publication history

The pre-publication history for this paper can be accessed here:

http://www.biomedcentral.com/1741-7015/11/148/prepub

## Note

This article is part of the cross journal collection *HIV 30 years on*. Other articles in this series can be found at http://www.biomedcentral.com/series/HIV_30.
